# Re-infection with SARS-CoV-2 is associated with increased antibody breadth and potency against diverse sarbecovirus strains

**DOI:** 10.1128/mbio.03612-25

**Published:** 2026-02-25

**Authors:** Michelle Lilly, Felicitas Ruiz, William B. Foreman, Vrasha Chohan, Jamie Guenthoer, Delphine Depierreux, Viren A. Baharani, Duncan Ralph, Alex Harteloo, Helen Y. Chu, Paul D. Bieniasz, Tyler N. Starr, Julie Overbaugh

**Affiliations:** 1 Human Biology Division, Fred Hutchinson Cancer Center 7286, Seattle, Washington, USA; 2 Molecular and Cellular Biology Graduate Program, University of Washington 7284, Seattle, Washington, USA; 3 Department of Biochemistry, University of Utah School of Medicine 12348 , Salt Lake City, Utah, USA; 4 Laboratory of Retrovirology, The Rockefeller University 5929, New York, New York, USA; 5 Laboratory of Molecular Immunology, The Rockefeller University 5929, New York, New York, USA; 6 Public Health Sciences Division, Fred Hutchinson Cancer Center 7286, Seattle, Washington, USA; 7 Division of Allergy and Infectious Diseases, University of Washington 7284, Seattle, Washington, USA; 8 The Rockefeller University, Howard Hughes Medical Institute 5929, New York, New York, USA; Duke University School of Medicine, Durham, North Carolina, USA

**Keywords:** SARS-CoV-2, affinity maturation, monoclonal antibodies, re-infection

## Abstract

The ease with which emerging SARS-CoV-2 variants escape neutralizing antibodies limits the protection afforded by a prior exposure, be it infection or vaccination. While rare, broadly neutralizing antibodies with activity toward diverse sarbecoviruses have been detected in convalescent serum. Motivated by findings that plasma responses show increased neutralization breadth and potency with continued antigen exposure, we isolated monoclonal antibodies (mAbs) after a SARS-CoV-2 re-infection and compared them to those isolated 1 year prior, after the first breakthrough infection. Among clonal lineage members identified at both time points, mAbs from the later time point showed improved neutralization potency and breadth. One mAb isolated after re-infection, C68.490, targets a conserved region in the receptor binding domain and shows remarkable activity not only against SARS-CoV-2 variants, but also diverse sarbecoviruses from more distant clades present in animal reservoirs. These findings suggest that a focus on individuals with diverse and repeated antigen exposure could lead to the identification of antibodies with therapeutic utility not just toward current and future SARS-CoV-2 variants, but also distant sarbecoviruses in the event of a future spillover.

**IMPORTANCE:**

Spillover of SARS-related viruses (sarbecoviruses) from animal reservoirs into humans has occurred multiple times in the past few decades. The most recent spillover due to SARS-CoV-2 continues to cause significant disease burden, and treatment options are few, in part because of selection for new variants due to immune escape. Thus, discovering antibodies that can block infection with sarbecoviruses, including SARS-CoV-2 variants, remains critical for both the current pandemic as well as those to come. Our study shows that an individual who was vaccinated and then had repeated breakthrough infections with distinct SARS-CoV-2 variants generated more potent antibodies after the second infection compared to the first infection. Notably, we discovered an antibody in this individual that not only neutralized the dominant SARS-CoV-2 variants but also a range of diverse sarbecoviruses present in animal reservoirs. This antibody thus holds promise as a therapeutic for both the current pandemic and future spillover events.

## INTRODUCTION

The SARS-CoV-2 pandemic has illustrated the devastating effects that can occur when a sarbecovirus originally from an animal reservoir is introduced into the human population. From this experience, we have come to appreciate the important clinical contribution of neutralizing antibodies to protection against sarbecovirus infections and disease severity. Neutralizing antibodies contribute to SARS-CoV-2 vaccine efficacy and are a correlate of protection from infection and disease (1). In addition, the therapeutic use of monoclonal antibodies (mAbs) against early strains of SARS-CoV-2 proved effective in decreasing disease severity, but efficacy waned when viral escape mutations arose (2–6). Neutralizing antibodies also showed promise against SARS-CoV-1, although they were not deployed due to the curtailment of that more limited sarbecovirus outbreak (7, 8). Characterizing neutralizing antibodies with broad sarbecovirus activity is therefore important for pandemic preparedness and prevention, given that sarbecoviruses are prevalent in a variety of animal reservoirs (9–12) and have recently seeded several human outbreaks.

Cross-reactive neutralizing antibodies that target functionally constrained epitopes across sarbecoviruses may also prove more durable against SARS-CoV-2 variation, which continues to compromise the efficacy of many neutralizing antibodies. Because the immune response generated by vaccination and infection is not sterilizing, the virus replicates for days, or sometimes weeks, in the face of neutralizing antibodies generated by these exposures. The most potent of these neutralizing antibodies typically target the receptor binding domain (RBD) in the S1 subunit of the viral spike glycoprotein (13–17). Not surprisingly, then, the antibody escape observed for SARS-CoV-2 variants is driven mostly by mutations in dominant RBD epitopes, which show remarkable ability to tolerate variation while retaining function (18–21). Unfortunately, this escape has occurred in epitopes targeted by therapeutic mAbs, rendering them ineffective and highlighting a need for mAbs that target more mutation-intolerant regions of the RBD (18, 22–24).

Sequence variation within RBD is not uniform, and some regions show very limited variation across circulating strains, suggesting there may be functionally constrained regions in the spike RBD. Indeed, several RBD-specific mAbs targeting more conserved regions that exhibit broad sarbecovirus neutralizing activity have been identified (22, 25–37). S2X259 was one of the first pan-sarbecovirus neutralizing antibodies characterized, although it rapidly lost activity against emerging SARS-CoV-2 Omicron variants (26, 37, 38). Subsequently, antibodies with impressive SARS-CoV-2 breadth have been isolated from individuals who were exposed to both SARS-CoV-1 and SARS-CoV-2. One notable example is SA55, which retains breadth and potency across all SARS-CoV-2 strains tested, including recent variants (26, 39). SA55 also exhibits binding and neutralizing capabilities against SARS-CoV-1, as well as a subset of sarbecoviruses from different clades (26, 38). A recently described potently neutralizing and cross-reactive antibody, VIR-7229, was obtained by mimicking the affinity maturation process through selection for mutations that led to improved binding *in vitro* (38). This mAb showed impressive binding breadth across all major SARS-CoV-2 variants and against all but a small subset of sarbecovirus spike proteins, although it exhibits more limited neutralization potency against some SARS-CoV-2 variants (38). There are examples of mAbs that are broadly neutralizing against SARS-CoV-2 variants and show some cross-reactivity toward distant sarbecoviruses, and conversely, there are mAbs that show cross-sarbecovirus breadth but limited SARS-CoV-2 neutralization, but there are few mAbs that do both (22, 26, 30, 35). Such antibodies, which would be predicted to target highly conserved and constrained epitopes, are likely to be relatively resistant to escape.

We previously identified several broadly neutralizing antibodies in one individual, C68, that experienced a heterologous antigen exposure to SARS-CoV-2 spike through a Delta post-vaccination infection (PVI) subsequent to vaccination with the ancestral WH-1 strain (36, 37). One of these mAbs (C68.61) targets a conserved region in the RBD where there has been minimal antigenic variation in SARS-CoV-2 variants (36) and escape could not be selected *in vitro* (37). Interestingly, C68.61 shows breadth not only across all SARS-CoV-2 variants tested, but also against a subset of sarbecovirus variants from animal reservoirs (37).

Given the resilient functional properties of mAbs from C68, we sought to determine whether there might be mAbs with even greater breadth or potency after subsequent antigen exposures by vaccinations and infection to genetically diverse SARS-CoV-2 variants in this individual. Several studies have examined plasma responses and shown increases in breadth and potency of neutralization with repeated exposure to heterologous spike antigen in polyclonal sera (40–43), although detailed studies of affinity maturation of individual monoclonal responses in the context of heterologous spike exposures are few (44, 45). Likewise, variation in SARS-CoV-2 may provide antigenic diversity that could drive the development of a more potent and/or broad neutralizing antibody response through somatic hypermutation (SHM) and affinity maturation. In line with this, individuals with both a WH-1 and an Omicron variant exposure developed higher and broader plasma neutralizing titers against Omicron variants than individuals with multiple exposures to the ancestral WH-1 strain by vaccination (18, 46–48).

To examine the process of affinity maturation of SARS-CoV-2-binding antibodies and identify cross-reactive neutralizing antibodies, we isolated spike-specific B cells from a later time point in the individual from whom C68.61 and other broadly active mAbs were isolated. We selected a sample obtained after an additional vaccination and a second PVI, which occurred during a period when Omicron variants were the major circulating strains. We characterized the genetic and functional properties of mAbs reconstructed from B cells that bound spike antigen, focusing on antibodies that were part of clonal lineage families to examine affinity maturation. We observed increased neutralization breadth and potency against SARS-CoV-2 variants in clonal lineage members isolated from this later time point compared to those from the earlier sample. Remarkably, we also identified a mAb, C68.490, from a clonal lineage not detected in earlier time points that showed breadth and potency against SARS-CoV-2 variants, as well as multiple sarbecoviruses representing several clades common in animal reservoirs.

## MATERIALS AND METHODS

### Participant information

A peripheral blood mononuclear cell (PBMC) sample collected from individual C68 in June 2022, 1 month after a second PVI, was used for this study. C68 had a first PVI with a Delta variant in July 2021, which was 2 months after immunization with two doses of the Pfizer-BioNTech mRNA vaccine. mAbs were previously isolated 1 month after this PVI (referred to here as the PVI-1 time point [49]). For the current study, the sample analyzed was 1 year later (PVI-2 time point), which was a month after a second infection, at a time when the Omicron BA.5 lineage was the dominant circulating variant, and 8 months after a third vaccination. All vaccinations were with the ancestral WH-1 strain. Study participant C68 was enrolled in the Hospitalized or Ambulatory Adults with Respiratory Viral Infections study.

### B cell isolation approach

The PBMC sample from PVI-2, which contained 11.2 million viable cells, was stained and sorted to isolate spike-specific memory B cells (MBCs) as previously described (36). In brief, cells were washed in FACS wash and stained with a cocktail of fluorescently-labeled antibodies to cell surface markers: CD3-BV711 (BD Biosciences, clone UCHT1), CD14-BV711 (BD Biosciences, clone MφP9), CD16-BV711 (BD Biosciences, clone 3G8), CD19-BV510 (BD Biosciences, clone SJ25C1), IgM-FITC (BD Biosciences, clone G20-127), IgD-FITC (BD Biosciences, clone IA6-2). PBMCs were also incubated with biotinylated SARS-CoV-2 Omicron XBB.1.5 spike trimer (Acro Biosystems, cat. SPN-C82Ez-25ug) and biotinylated SARS-CoV-1 spike trimer (Acro Biosystems, cat. SPN-S82E3-25ug; CUHK-W1 strain, accession #: AY278554.2). Biotinylated spike proteins were conjugated to streptavidin-fluorophores APC and PE (from BioLegend) overnight at 4°C, the day before cell sorting. PBMCs were loaded onto a BD FACS Aria II cell sorter, and IgG-expressing, spike-specific B cells were identified as CD3−, CD14−, CD16−, CD19+, IgD−, IgM−, PE+, APC+ and live cells (Ghost Dye Red 780; Tonbo Biosciences cat: 13-0865-T500) and were sorted into a total of four 96-well PCR plates containing cell lysis/RNA storage buffer and stored at −80°C. A total of 384 SARS-CoV-2 Omicron XBB.1.5 spike trimer and/or SARS-CoV-1 spike trimer-specific B cells were recovered from this sample.

### Recombinant antibody production and purification

Amplification of variable heavy and light chain genes (VH and VL, respectively) was performed as described (36, 50, 51). In order to identify clonal lineage members, the 384 B cells that bound spike were first subject to PCR to amplify the VH gene fragment, and the sequence of the resulting PCR products was determined. A total of 269 VH gene sequences were successfully amplified, with 254 predicted to encode productive VH genes based on the presence of a variable, diversity, and joining (VDJ) gene rearrangement with an in-frame open reading frame and with no premature stop codons.

To identify the subset that was members of the same clonal lineage, clonality was assessed based on VDJ gene segment rearrangement using partis, a BCR sequence annotation software package designed for antibody sequence analysis (52, 53). This analysis was performed for the 269 PVI-2 VH sequences, of which 254 were predicted to encode productive genes, combined with 118 PVI-1 VH sequences from prior studies, which included 100 VH genes that were further functionally characterized (36, 37, 49). When clonal lineages were identified based on the VH sequence, either within the PVI-2 sequences or between PVI-2 and PVI-1 sequences, the VL chain sequence was amplified from select MBCs. Specifically, as a means to focus on those with known neutralizing activity, in cases where PVI-2 antibodies were clonal to a PVI-1 antibody, the lineages where the PVI-1 antibody was RBD-specific were pursued further. In cases where there were clonally related VH sequences within PVI-2 but no PVI-1 clonal counterparts, we rescued the VL gene corresponding to the antibody sequence with the highest SHM in the VH gene as defined by partis (52, 53). In cases where the antibody with the highest SHM could not be isolated or cloned, another antibody from the same clonal lineage was selected, if available.

After the isolation of VL genes of interest, sequences were again assessed for clonality using partis in order to improve clonal lineage determinations with the addition of paired heavy-light chain sequences (53). This second determination, based on both VH and VL sequences, was used in the final analysis. Based on the secondary partis determination, a total of 33 clonal lineages were identified, of which 22 had lineage family members within PVI-2 only, and 11 had lineage family members in both PVI-2 and PVI-1.

mAbs were generated by transfecting FreeStyle 293-F Cells (Invitrogen) with paired VH and VL chains as described (36). The resulting mAbs were purified by affinity chromatography using protein G agarose columns (Pierce).

### ELISA binding assay

Antibody binding to SARS-CoV-2 or SARS-CoV-1 recombinant spike protein was assessed using ELISA binding assays as previously described (54). All antibodies were tested at a single concentration (1,000 ng/mL), and OD450 nm values were measured as a qualitative test of binding. The raw OD450 values were averaged between technical duplicates and background subtracted with the negative control wells treated with HIV mAb VRC01. All described antibodies were tested for binding to SARS-CoV-2 WH-1 RBD (Sino Biological, cat. 40592-V08H), SARS-CoV-2 WH-1 S1 (Sino Biological, cat. 40591-V08H), SARS-CoV-2 XBB.1.5 trimer (40589-V08H45), and SARS-CoV-1 GD01 trimer (Acro Biosystems, cat. SPN-S52Ht). In addition, a subset was also tested for binding to SARS-CoV-2 Delta trimer (Sino Biological, cat. 40589-V08H10) and SARS-CoV-2 B.Q.1.1 trimer (Sino Biological, cat. 40589-V08H41).

### Neutralization assay

Antibodies were tested for neutralization using spike-pseudotyped lentiviral particles and 293T-ACE2 expressing cells (55) in a 384-well format, as described previously (36). Sarbecovirus spike plasmids with spike genes specific for SARS-CoV-2 variants, SARS-CoV-1 (Urbani strain), BtKY72 (K493Y/T498W mutant), Khosta-2, WIV1, LYRa3, Pangolin-GD, and RsSHC014 were generated as previously described (36, 48, 49). Antibody dilutions were prepared at a starting concentration of 20 μg/mL, with threefold dilution in a total of six dilutions assessed, with the exception of C68.490, and control antibodies C68.61 and S2X259, where twofold mAb dilution curves for a total of 12 dilutions were used. The inhibitory concentration at 50% (IC50) was calculated with GraphPad PRISM statistical software by fitting a four-parameter (agonist vs response) non-linear regression curve with the bottom fixed at 0, the top constrained to 1, and HillSlope <0. In cases where fraction infectivity did not cross 0.5, the experiment was repeated with a lower starting mAb concentration so that the neutralization profile encompassed a sigmoidal curve with data points above 50% infectivity.

### Antibody binding to yeast-displayed pan-sarbecoviruses RBD

To evaluate the ability of C68.490 to bind a panel of sarbecovirus RBDs, we used yeast libraries displaying RBDs from sarbecoviruses representing different clades as previously described (38). A full list of all RBDs and sequence accession numbers is available at https://github.com/tstarrlab/SARSr-CoV_mAb-breadth_Overbaugh/blob/main/data/sarbeco_accessions.csv. Each sarbecovirus RBD is represented by multiple (often >100) barcodes such that technical pseudo-replicates can be ascertained within each binding experiment. C68.490 was incubated with a sarbecovirus-RBD-displaying yeast library at five concentration points (10,000 ng/mL and four serial 25-fold dilutions to 0.0256 ng/mL; plus zero mAb condition). Cells displaying RBDs were sorted via FACS into 1 of 4 bins according to binding toward C68.490. To set these bins, a known-binding pair of isogenic yeast-displayed RBD and antibody was incubated at saturation (10,000 ng/mL) and 0 ng/mL (no antibody). Bins were drawn such that bin 1 captured 95% of cells in the 0 ng/mL population, and bin 4 captured 95% of cells in the 10,000 ng/mL population. Barcodes were counted within each FACS bin via Illumina sequencing, with raw barcode counts available at https://github.com/tstarrlab/SARSr-CoV_mAb-breadth_Overbaugh/blob/main/results/counts/variant_counts.csv. EC50s were calculated by fitting the Hill equation (where *n* = 1) to bin values 1 to 4 for each barcoded sarbecovirus RBD across the antibody concentration series, allowing us to compare an antibody’s affinity toward sarbecovirus RBDs in the library. EC50 scores are the geometric mean across the independent barcodes. The computational pipeline for computing sarbecovirus mAb-binding breadth is available from https://github.com/tstarrlab/SARSr-CoV_mAb-breadth_Overbaugh.

### Epitope profiling by deep mutational scanning of the RBD

To determine the epitope targets and the escape profile of the C68.490 mAb, we used a yeast library displaying RBD proteins with nearly every single amino acid mutation in the RBD of SARS-CoV-2 Wuhan-Hu-1, SARS-CoV-2 Omicron BA.2, and SARS-CoV-1 Urbani as previously described (14, 36, 37). A FACS-based approach was used to identify antibody escape mutants by gating on yeast that display RBD mutants based on antibody binding, with gates drawn on wild-type control cells labeled at 10% of the library selection mAb concentration to approximate an escape bin of 10× or greater loss of binding (Fig. S1A), sorted on a BD FACS Aria II.

Pre-sort and sorted antibody-escape cells were sequenced using an Illumina NovaSeq, and a 16-nucleotide barcode linked to each RBD mutant was used to identify the mutant sequence. Raw sequence read counts are available from GitHub: https://github.com/tstarrlab/SARS-CoV-2-RBD_Omicron_MAP_Overbaugh-C68-490/tree/main/results/counts. Sequencing reads were then compiled and compared to the pre-sort population frequencies to generate the “escape fraction” visualized on line plots (Fig. S1B). Experiments were performed in biological duplicate using independent mutant RBD libraries that correlate well (Fig. S1C), so escape fractions represent the average of these two independent biological replicates. Final escape fraction measurements averaged across two replicates are available from https://github.com/tstarrlab/SARS-CoV-2-RBD_Omicron_MAP_Overbaugh-C68-490/tree/main/results/supp_data. The entire pipeline for epitope escape profiling is available from https://github.com/tstarrlab/SARS-CoV-2-RBD_Omicron_MAP_Overbaugh-C68-490.

### Escape mutations identified by replication-competent recombinant virus surface-expressed spike protein

To assess which escape mutants arise in the presence of C68.490 mAb in the context of virus replication, we used a recombinant replication-competent vesicular stomatitis virus (rVSV) expressing the SARS-CoV-1 spike protein (21). The methods used were similar to those described previously (37), including the generation of virus, infections, and Illumina sequencing methods of virus output, which was harvested at 48 h after the second passage. mAb C68.490 was tested at two concentrations, 50 and 250 ng/mL, against rVSV expressing SARS-CoV-1 (CUHK-W1).

## RESULTS

### Isolation of spike-specific monoclonal antibodies after re-infection

**Fig 1:**
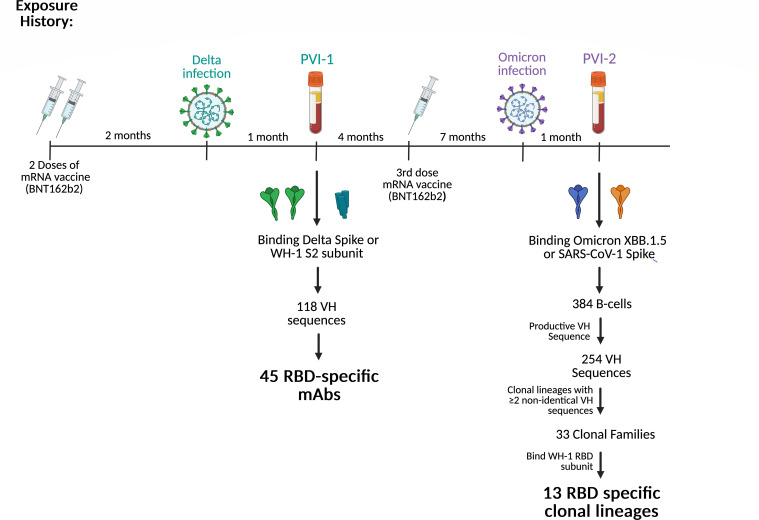
Schematic of individual C68’s exposure history with timing and approach for antibody isolation. A timeline indicating intervals of vaccination and infection is shown at the top. The time points at which antibodies were isolated (PVI-1 and PVI-2) are indicated. Below the timeline, the antigens used to select antigen-specific memory B cells are shown. The process used to identify the 13 RBD-specific antibodies from PVI-2 used for downstream studies is shown to the right. Created in BioRender (M. Lilly, 2026, https://BioRender.com/qo0myte).

To examine the evolution of the antibody response in C68 after additional exposures to SARS-CoV-2 antigen compared to our first sampling (49), we isolated spike-specific MBCs from peripheral blood mononuclear cells collected at 1 month after the second PVI (sample PVI-2), which was during a period when Omicron BA.5 was the dominant circulating variant. This individual previously experienced multiple SARS-CoV-2 antigen exposures, including a first PVI with the Delta variant 1 year prior and an intervening vaccination (WH-1) 7 months prior to the second infection (Fig. 1). MBCs that bound spike were isolated from the PVI-2 sample using SARS-CoV-2 Omicron XBB.1.5 and SARS-CoV-1 (CUHK-W1 strain) trimers (Fig. 1). These two spike proteins were chosen to enrich for antibodies with broad activity against SARS-CoV-2 and/or SARS-CoV-1.

To identify clonal lineages, we pooled VH sequences at the PVI-2 time point with those previously isolated from the PVI-1 time point 1 year prior. We identified 33 cases of expanded clonal lineages (Fig. 1), which we defined as at least two non-identical VH members. Of those, 11 were clonal lineage families that were detected in both PVI-2 and PVI-1 samples, and 22 were new clonal lineage families only found in the PVI-2 sample. Because most potently neutralizing antibodies target the RBD, this subset was further selected for those that bound to RBD by ELISA. This resulted in a total of 13 clonal lineages that target the RBD: four clonal lineages that include both PVI-1 and PVI-2 members and nine that were identified only in the PVI-2 sequences for downstream studies.

### PVI-2 antibodies show increased binding and neutralizing activity compared to clonally related PVI-1 antibodies

**Fig 2:**
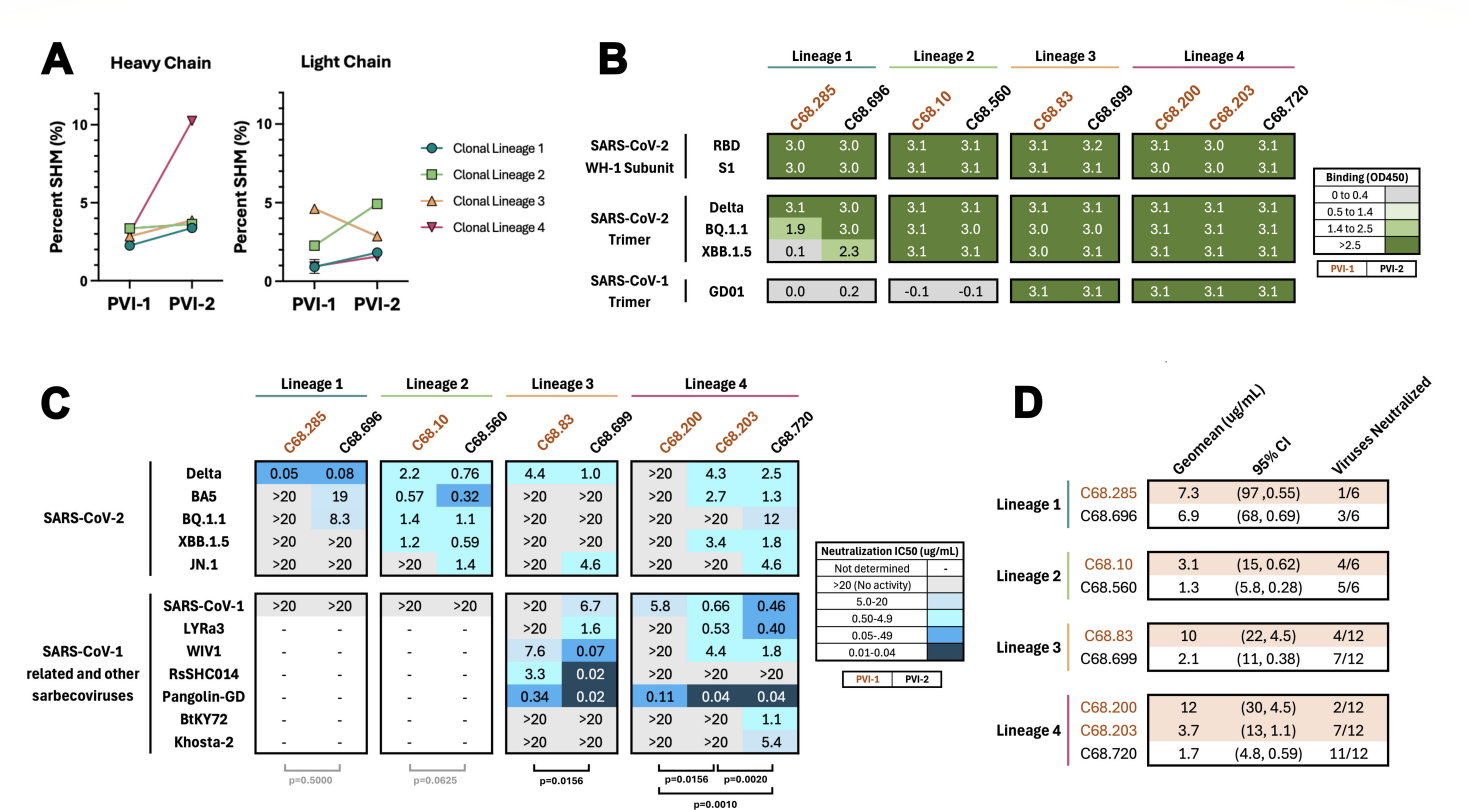
Functional activity of PVI-2 antibodies compared to PVI-1 antibodies from the same clonal family. (**A**) Percent somatic hypermutation of IgG heavy chain and light chain among clonal lineage members. (**B**) Comparison of binding profiles between PVI-1 mAbs and clonal lineage members in PVI-2. The heatmap shows OD450 nm binding activity by ELISA, with the antigens tested shown to the left. The antibodies tested are indicated at the top with mAbs from PVI-1 in orange text and PVI-2 in black text. mAbs were tested in duplicate at a fixed concentration of 1,000 ng/mL. Green indicates binding with increasing shades of green indicating increasing OD450 values, as indicated in the key to the right. The cases with the darkest green (>3.0 OD450) are qualitative and not quantitative results, as the assay would be saturated for the most potent binding mAbs. Gray indicates no detectable binding. (**C**) Heatmap of neutralization IC50s (µg/mL) of the same antibodies as in panel B. Viruses tested are shown to the left, with SARS-CoV-2 variants in the top half and more diverse sarbecoviruses in the bottom half. The color gradient represents neutralization activity as shown in the key to the right, with darker shades of blue correlating with IC50 potency. Gray indicates no detectable neutralization (IC50), and white indicates the mAbs were not tested because they did not bind SARS-CoV-1 spike trimer. IC50 values were averaged from 2 to 6 independent experiments performed in technical duplicate. IC50 values were calculated with GraphPad Prism, with a four-parameter non-linear regression model. (**D**) Geometric mean of the IC50 values (µg/mL) and 95% confidence intervals (CI) of the panel of viruses tested in panel **C**. IC50 >20 µg/mL was set to 20 µg/mL in this calculation. The number of viruses neutralized is displayed as a fraction of viruses that the antibody neutralizes with an IC50 <20 µg/mL, over the total number of viruses the antibody was tested against.

To address whether re-infection and associated continued antigen exposure impact antibody breadth and function, we first focused on the four clonal lineages with antibodies from both PVI-1 and PVI-2. All four PVI-2 mAbs had higher levels of SHM than PVI-1 mAbs from the same clonal lineages based on VH chain sequences, but only clonal lineage 4 showed a notable change in SHM of 7.1% (from 3.1% to 10.2%). For the other three clonal lineages, increases in SHM were more modest, ranging from 0.2% to 1.1% (Fig. 2A). Changes in SHM were similarly modest for three of the VL sequences (ranging from an increase of 0.7% to 2.6%), with one lineage (clonal lineage 3) having a decrease in SHM from 4.6% to 2.9%.

The binding breadth of these mAbs was examined by ELISA against several spike proteins, including those from the ancestral WH-1 strain, as well as SARS-CoV-2 variants and SARS-CoV-1. All PVI-2 mAbs bound WH-1 RBD and S1 subunits, as well as Delta trimer (Fig. 2B). One PVI-2 mAb, C68.696, showed increased binding breadth against Omicron variants relative to the PVI-1 mAb, C68.285, from the same clonal lineage (clonal lineage 1). In this case, C68.696 acquired the ability to bind the SARS-CoV-2 XBB.1.5 trimer compared to the related PVI-1 mAb. For the other three clonal lineages, the binding patterns were similar, and for clonal lineages 3 and 4, both PVI-1 and PVI-2 mAbs bound all spike antigens tested.

We next compared the neutralization activity between these clonal mAbs against a small panel of SARS-CoV-2 variants, including Delta and Omicron variants (Fig. 2C). In general, PVI-2 mAbs showed similar or increased neutralization (i.e., lower IC50) compared to their clonal lineage members from the PVI-1 time point. PVI-2 mAbs from all four clonal lineages neutralized at least one virus more potently than their PVI-1 clonal counterparts, although in most cases, the differences were subtle (<2-fold). The exception was JN.1, where PVI-1 mAbs did not show detectable neutralization, but PVI-2 mAbs did.

The PVI-2 mAbs of clonal lineage families 3 and 4 neutralized SARS-CoV-1. For these clonal lineages, we further examined neutralization activity against a larger panel of sarbecoviruses. We pseudotyped viruses with spikes from representative viruses from three clades of sarbecoviruses. This included Pangolin-GD, a clade 1b virus closely related to SARS-CoV-2 (96% sequence identity to the ancestral SARS-CoV-2 RBD), and several clade 1a viruses related to SARS-CoV-1, including WIV1 (96% sequence identity to ancestral SARS-CoV-1 RBD), LYRa3 (95%), and RsSHC014 (82%). In addition, we tested two more distantly related viruses from clade 3 sarbecoviruses that include isolates from Africa and Europe, BtKY72 and Khosta-2 (73% and 68% to SARS-CoV-2 RBD, respectively). Because wild-type BtKY72 has reduced binding of human ACE2, we utilized a K493Y/T498W mutant that confers entry via human ACE2 (56).

In both clonal lineages, PVI-2 mAbs showed increased breadth and potency in neutralization against this panel of sarbecoviruses compared to PVI-1 mAbs. For instance, in clonal lineage 3, PVI-2 mAb C68.699 neutralized five of the seven sarbecoviruses tested compared to three of seven for the PVI-1 clonal lineage member (C68.83). These mAbs also showed differences in potency; for example, the PVI-2 mAb neutralized RsSHC014 with more than a 100-fold greater potency compared to its PVI-1 clonal lineage member (IC50 = 3.3 vs 0.02 µg/mL). Similar increases in breadth and potency in sarbecovirus neutralization were also seen for clonal lineage 4. In this case, the two sarbecoviruses with the lowest sequence similarity to SARS-CoV-2, BtKY72 and Khosta-2, were not neutralized by either mAb from the PVI-1 time point, but both were neutralized by the PVI-2 mAb C68.720.

While there was not a statistically significant difference in neutralization IC50s between PVI-1 and PVI-2 mAbs (Wilcoxon matched-pairs signed rank test, *P* = 0.5 and *P* = 0.0625, respectively), there was significantly more potent neutralization for lineages 3 and 4 PVI-2 versus PVI-1 mAbs (*P* = 0.0156 and *P* > 0.0156, respectively). Overall, the PVI-1 mAbs from all four clonal lineages displayed lower geometric mean IC50s (Fig. 2D) than the PVI-2 mAbs across the panel of SARS-CoV-2 and sarbecovirus variants. In each case, the number of viruses neutralized was also greater for PVI-2 mAbs when compared to those isolated a year prior at PVI-1, including against sarbecoviruses that were not used to select for antigen-positive B cells or a known part of the patient’s exposure history.

### Characterization of antibodies representing PVI-2-specific clonal lineages

In addition to clonal lineage families represented in both PVI-1 and PVI-2, we also identified nine RBD-specific clonal lineages that were unique to PVI-2. For two lineages (6 and 13), two mAbs were included, resulting in a total of 11 RBD-specific antibodies. These clonal lineages utilized a diversity of VDJ genes. However, light chain gene usage was more homogenous, with five of nine clonal lineages utilizing IGVK1-5 (Fig. S2).

**Fig 3:**
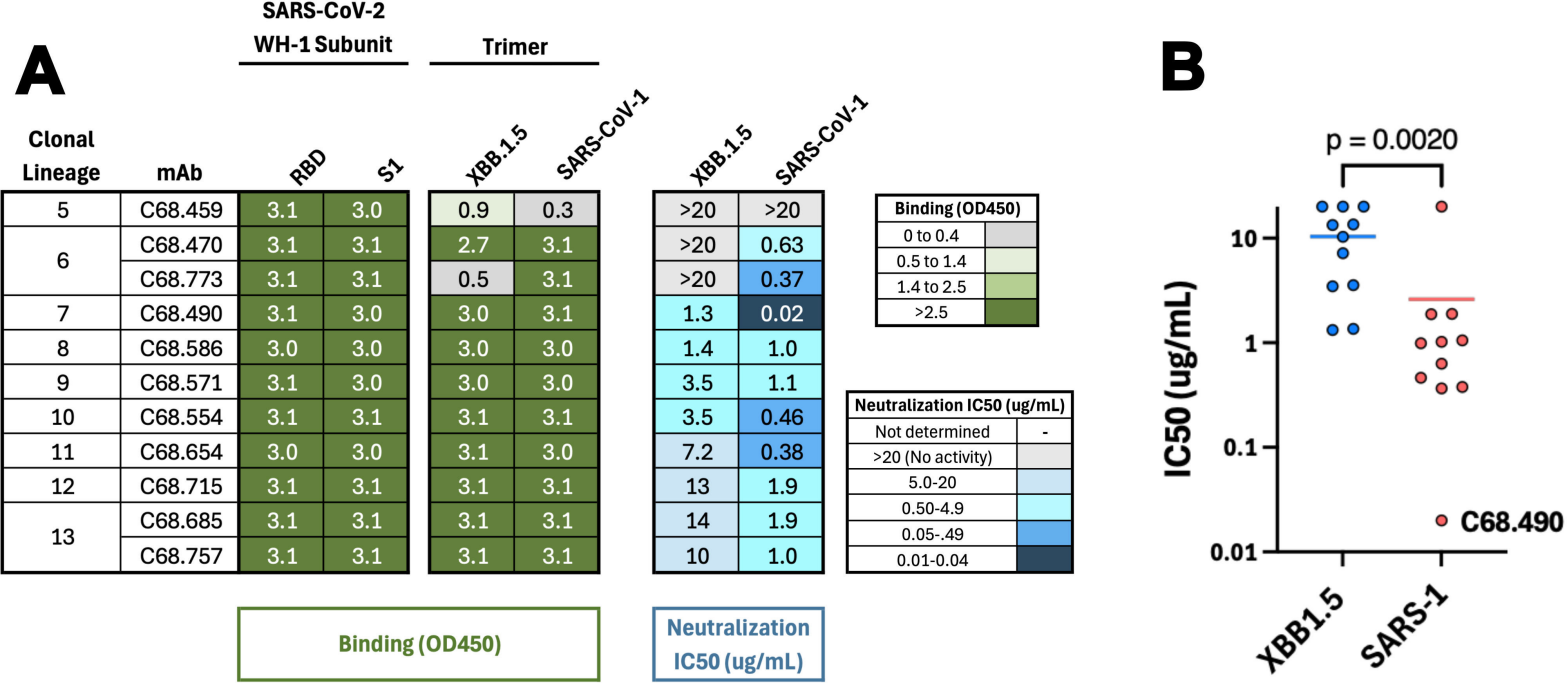
Binding and neutralization of antibodies representing new clonal lineages specific to PVI-2. (**A**) Heatmap shows binding (green) and neutralization IC50s (blue, last two columns). Viruses tested are shown at the top, and mAbs are indicated to the left, after the clonal family designation. IC50 values were averaged from two independent experiments performed in technical duplicate. Figure details are as described in Fig. 2B and C. (**B**) Comparison of IC50s for SARS-CoV-2 XBB1.5 and SARS-CoV-1 for antibodies from PVI-2 specific clonal lineages. The *P* value is calculated from the Wilcoxon matched-paired signed rank test. Antibodies with no neutralization activity (IC50 > 20 µg/mL) were set to 20 µg/mL for this comparison.

All 11 PVI-2 mAbs bound both SARS-CoV-2 WH-1 RBD and S1 subunits (Fig. 3A). The majority bound XBB.1.5 and SARS-CoV-1 spike, with only 2 of 11 showing no or only weak binding to one or more spike variants. We assessed the ability of these mAbs to neutralize viruses pseudotyped with spikes from SARS-CoV-2 XBB.1.5 and SARS-CoV-1, which are the spike proteins used for isolation of MBCs from PVI-2. Most of the antibodies (8/11) had detectable neutralization (IC50 < 20 µg/mL) against both XBB.1.5 and SARS-CoV-1 pseudoviruses, and all but C68.459 were capable of neutralizing at least one of these viruses. IC50s ranged from 1.3 to >20 µg/mL (mean IC50 = 10 µg/mL) against SARS-CoV-2 XBB.1.5. Interestingly, in all cases, except C68.459, these mAbs more potently neutralized SARS-CoV-1 than XBB.1.5 (Fig. 3B), with IC50s ranging from 0.02 to >20 µg/mL (mean IC50 = 2.6 µg/mL). The most notable of these mAbs was C68.490, which neutralized SARS-CoV-1 at least 10-fold more potently than any other tested PVI-2 mAb (IC50 = 0.02 µg/mL).

### C68.490 demonstrates potent binding and neutralization across diverse sarbecovirus clades

**Fig 4:**
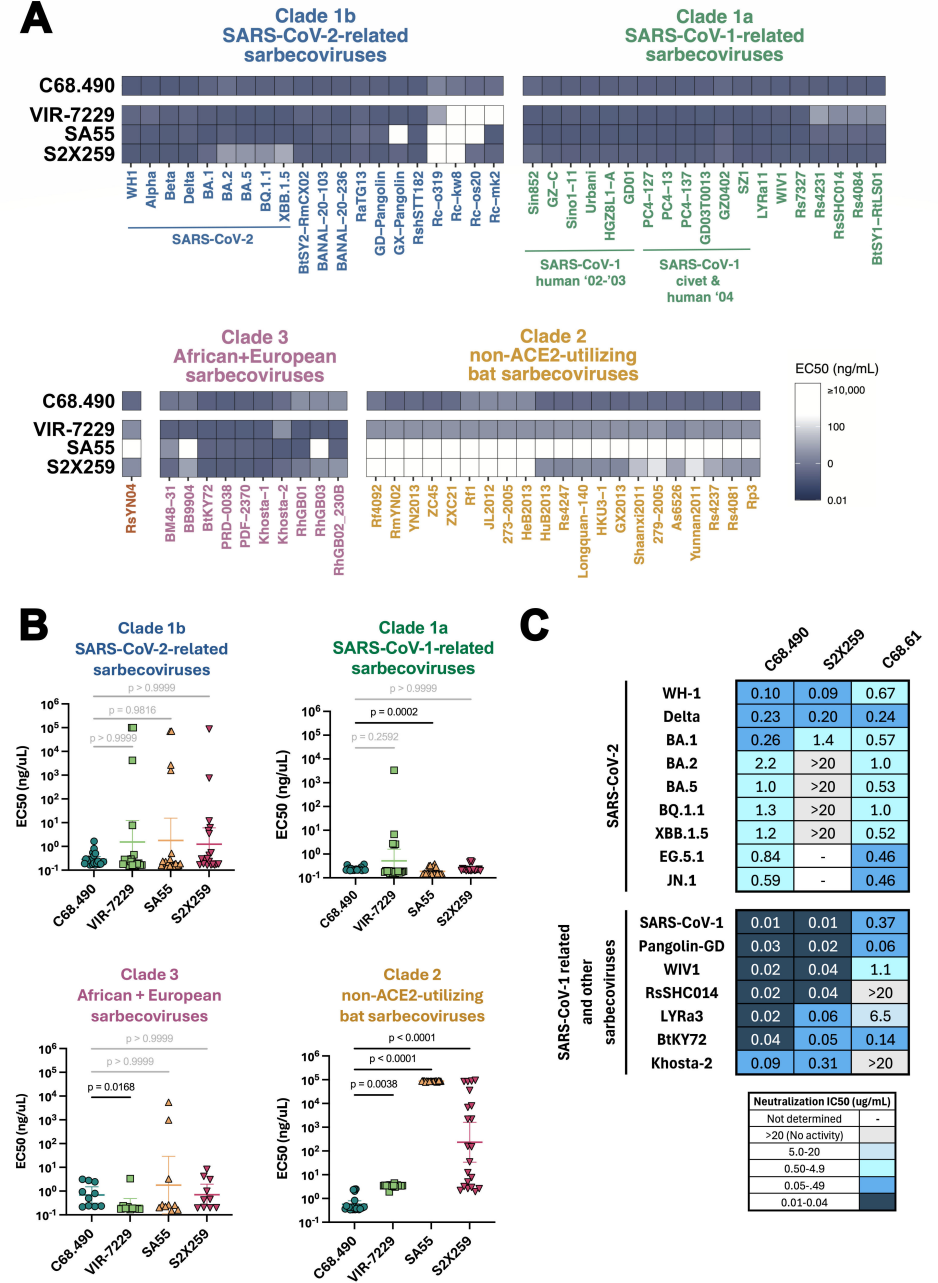
Functional characterization of a pan-sarbecovirus neutralizing antibody C68.490. (**A**) Binding breadth of C68.490 and previously characterized antibodies with known pan-sarbecovirus activity against a library of yeast-display sarbecovirus RBDs. Antibodies tested are shown to the left with the specific RBD indicated at the bottom. RBDs are classified by clades as shown above and color-coded. Data for VIR-7229, SA55, and S2X259 were previously published by reference 38 and shown for comparison. (**B**) Comparison of C68.490 binding with other pan-sarbecovirus antibodies by sarbecovirus clade. Statistically significant differences in mean EC50 values between C68.490 and other antibodies were assessed using the Friedman test with Dunn’s multiple comparisons test. Sarbecovirus RBDs were assigned to clades based on existing clade definitions (14). (**C**) Neutralization of SARS-CoV-2 and sarbecovirus variants by C68.490. Antibodies tested are described at the top and include two control mAbs for comparison. The viruses tested are shown to the left, with a line separating SARS-CoV-2 variants from the more diverse sarbecoviruses. Neutralization data are represented as IC50s (µg/mL) and color-coded as shown in the table below. IC50 values were averaged from 2 to 4 independent experiments performed in technical duplicate, with the exception of neutralization of S2X259 against SARS-CoV-1, which was only tested once. Data for C68.61, which was tested in parallel to the C68.490, was also reported in reference 37. Other details are as in Fig. 2C.

Given that C68.490 showed potent neutralization activity against SARS-CoV-1 and also neutralized SARS-CoV-2 XBB.1.5, we sought to determine if this activity extended to other sarbecoviruses and additional SARS-CoV-2 variants. As a first step, we profiled binding breadth against a panel of 72 sarbecovirus RBDs using a yeast display system (27, 38). This library includes multiple variants within each sarbecovirus clade (clades 1a, 1b, 2, and 3) (27, 38) and thus includes RBDs from ACE2 receptor-utilizing sarbecoviruses, similar to SARS-CoV-1, SARS-CoV-2, and BtKY72, as well as RBDs from more divergent ACE2-independent viruses (clade 2). Remarkably, C68.490 bound the RBD of all 72 tested sarbecoviruses with EC50s less than 10 ng/mL, including potent binding to RBDs from the divergent non-ACE2-utilizing bat sarbecovirus clade (Fig. 4A). Notably, the binding was broader than several of the most cross-reactive RBD mAbs previously described, including S2X259 (22, 25), SA55 (18, 24, 39), and VIR-7229 (38) (Fig. 4A). Among those mAbs, VIR-7229 showed the greatest breadth, with detectable binding to the RBD of all but a few members of the clade 1b family. C68.490 binding extended to all clade 1b RBDs and notably showed stronger binding to the clade 2 RBDs compared to VIR-7229 (Fig. 4B); the other mAbs showed no (SA55) or limited (S2X259) detectable binding to RBDs from this clade. C68.490 binding was not statistically different from other antibodies against clade 1b RBDs. Binding was slightly less compared to SA55 (mean EC50 = 0.24 vs 0.20 ng/uL) for clade 1a RBDs and compared to VIR-7229 for clade 3 RBDs (mean EC50 = 1.2 vs 0.51 ng/uL).

To determine if C68.490 also demonstrated breadth in neutralization, we determined the neutralization IC50s of this antibody against major and recently circulating SARS-CoV-2 variants, as well as the ACE2-dependent sarbecoviruses described above. Among SARS-CoV-2 variants tested, C68.490 neutralized all viruses with IC50 measurements ranging from 0.10 to 2.2 µg/mL (Fig. 4C). While there was slightly reduced neutralization in BA.2 sublineage variants (IC50 = 0.59–2.2 µg/mL), in comparison to pre-BA.2 variants (IC50 0.1–0.26 µg/mL), C68.490 retained neutralization against more recently circulating variants EG.5.1 and JN.1 (IC50 = 0.84, 0.59), similar to C68.61, which was tested in parallel as an example of a mAb notable for its breadth against SARS-CoV-2 variants (36, 37).

C68.490 potently neutralized SARS-CoV-1 and other tested sarbecoviruses, with IC50s ranging between 0.01 and 0.09 µg/mL. C68.490 was ~37-fold more potent than C68.61, which is an RBD-specific mAb isolated from PVI-1 that exhibits considerable sarbecovirus breadth (36, 37). Notably, C68.490 potently neutralized viruses with relatively low sequence similarity to SARS-CoV-2, like BtKY72 (IC50 = 0.04 µg/mL) and Khosta-2 (IC50 = 0.09 µg/mL). The breadth and potency of C68.490 against this sarbecovirus panel were similar to that of S2X259, which is one of the broadest cross-sarbecovirus mAbs yet described (25). Thus, overall, C68.490 is notable for its broad and cross-reactive neutralizing activity against both SARS-CoV-2 variants and diverse sarbecoviruses, consistent with its broad binding profile.

### C68.490 targets a highly conserved RBD epitope with limited escape pathways

**Fig 5:**
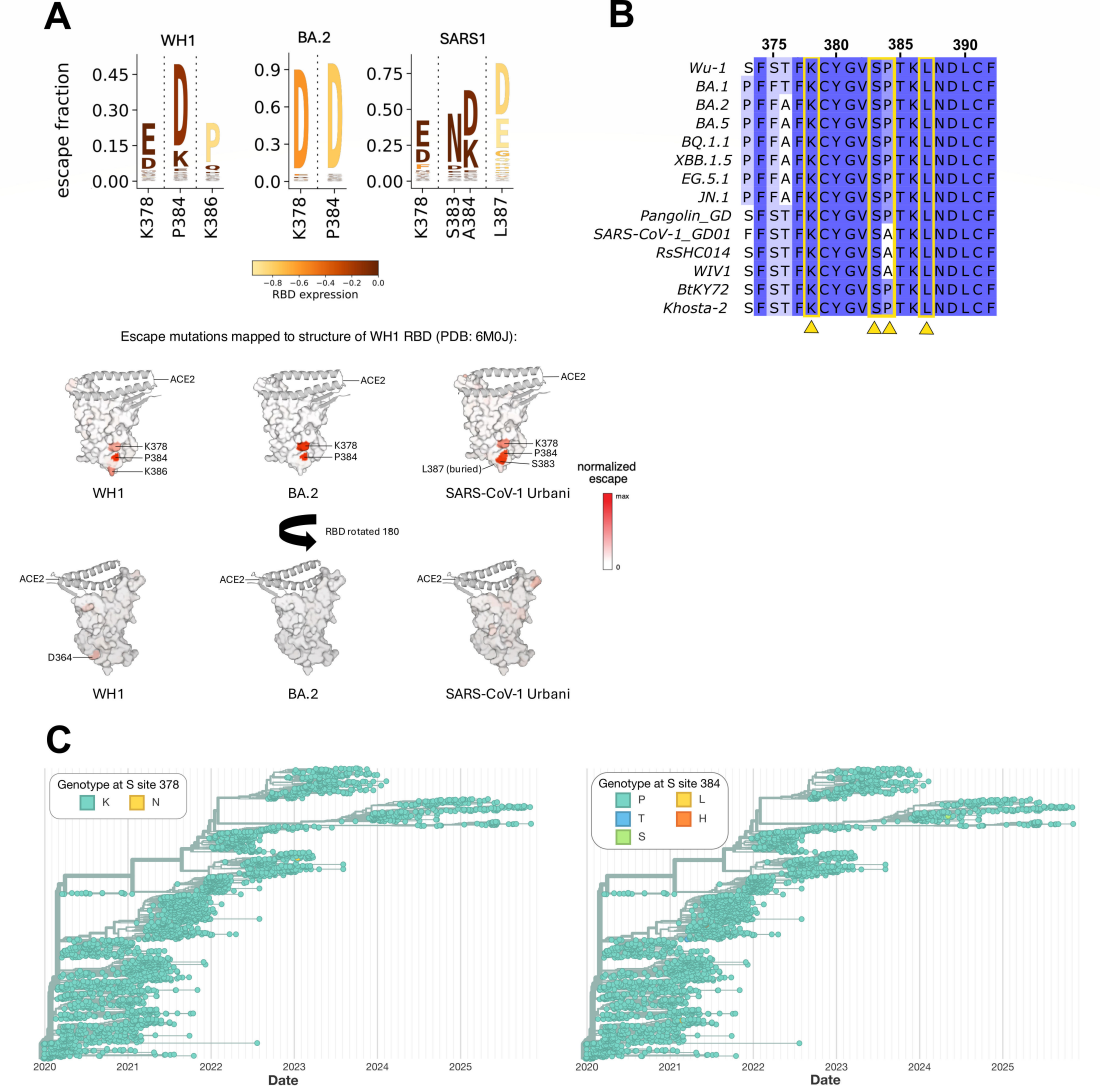
Characterization of C68.490 escape mutations. (**A**) Top, sites of binding escape and residues conferring escape from C68.490 in different viral backgrounds (WH1 = SARS-CoV-2 Wuhan-Hu-1, BA.2 = SARS-CoV-2 Omicron BA.2, SARS1 = SARS-CoV-1 Urbani) by deep mutational scanning of the RBD. Amino acid numbering based on the SARS-CoV-2 WH1 sequence. Bottom, sites of escape mapped onto surface representation of the RBD (key interacting motifs of ACE2 shown as the gray ribbon). Color gradient represents the escape fraction, with darker red encompassing degree of escape. (**B**) Multi-clade sarbecovirus sequence alignments. Conserved sites (in blue) and variable residues (in white) across sarbecoviruses around the epitope of C68.490 defined in panel **A** are depicted. C68.490 sites of escape are indicated with yellow arrows. (**C**) Genotype at SARS-CoV-2 spike amino acid sites 378 and 384 from 2,967 GenBank sequences. Adapted from Nextstrain.org (retrieved 16 November 2025).

To define the epitope of C68.490, we examined binding to a deep mutational scanning (DMS) library encoding virtually all single mutations within the RBD, allowing the identification of the functional epitope (57) as sites where mutations escape antibody binding. Because the epitope profiling can be influenced by epistatic interactions with other amino acid residues (21, 58), binding was assessed using DMS libraries in the background of two SARS-CoV-2 variants, WH-1 and BA.2 (58), as well as, for the first time, SARS-CoV-1 (59). For all three RBDs, the epitope of C68.490 was focused within sites 378-387, based on WH-1 numbering, with two shared binding escape sites, positions 378 and 384, across all three RBD proteins (Fig. 5A). There were also unique escape sites in some backgrounds, such as mutations at 386 that led to reduced mAb binding for WH-1 and mutations at 383 and 387 that escape binding in SARS-CoV-1. A common feature of mutations that were most disruptive to binding across all three RBDs was a change to a negatively charged amino acid. At position 384, aspartic acid (D) was the most enriched amino acid escape mutation across all three RBDs.

Several of the mutations that reduced binding of C68.490 to the RBD also reduce RBD expression, as defined in previous studies using these yeast RBD libraries to examine mutant effects on antibody escape and ACE2 binding (19, 58, 59) (Fig. 5A, yellow color). This is most notable in the BA.2 background where both dominant escape mutants also reduce RBD expression, indicating that while these mutations reduce binding of C68.490, viruses encoding these may have impaired fitness due to poor RBD folding. Indeed, when we performed experiments using replication-competent recombinant VSV-G encoding SARS-CoV-1 CUHK-W1 spike, we saw little selection for escape. In this case, the spike tested corresponded to the most potently neutralized pseudovirus in our panel, and thus was under pressure to escape. At a concentration of 50 ng/mL, no escape was observed, whereas at a concentration of 250 ng/mL, there was only a minor fraction (14%) of the virus that had a mutation, and this was at a position, G381R, outside of the core epitope.

Sequence alignment of all escape sites identified in the DMS escape profiles illustrates that these sites are highly conserved across both SARS-CoV-2 variants, as well as other sarbecovirus sequences. Three out of four escape sites are completely conserved across all the viruses tested for neutralization (Fig. 5B). At the fourth site, SARS-CoV-1, RsSHC014, and WIV1 encode an alanine at position 384 compared to a proline in the other RBDs. However, this amino acid substitution does not disrupt binding (Fig. 5A), which is consistent with the finding that the presence of alanine 384 was not associated with a reduction in neutralization activity (Fig. 4C).

In an analysis of 2,967 geographically and temporally diverse SARS-CoV-2 genomes from GenBank at site K378 using Nextstrain (60), only one mutation was observed (Fig. 5A). At site P384, four mutations have been rarely observed. None of these mutations was predicted to lead to binding escape in any of the three viral backgrounds tested by DMS.

## DISCUSSION

Given the prevalence of sarbecoviruses in animal reservoirs and their demonstrated capacity to enter the human population, continued spillover events and associated pandemics are possible. The rapid emergence of variants during the SARS-CoV-2 pandemic illustrated that once the virus circulates in humans, there is rapid immune escape driven by mutations in the spike protein. Thus, to effectively target these viruses, antibodies must target conserved regions of the viral spike protein and have broad neutralizing activity. Here, we built on studies suggesting that multiple exposures to diverse SARS-CoV-2 spike antigens can lead to broad plasma antibody responses (40–43) by examining this response at the monoclonal level. mAbs isolated after re-infection showed increased breadth compared to those from the same clonal lineage isolated after the first infection with a different strain. Notably, the mAbs isolated after re-infection showed neutralizing activity against both SARS-CoV-2 and other sarbecoviruses, with one, C68.490, showing remarkable breadth against diverse sarbecoviruses. Thus, these mAbs, particularly C68.490, may be a valuable tool for the prevention of or response to future sarbecovirus spillovers.

While studies of plasma antibody responses have shown that continued antigen exposure with diverse strains can lead to broader responses (40–43), little was known about the details of this response. By comparing members of the same clonal lineage after a first breakthrough with the Delta variant to those present after a second breakthrough with an Omicron variant, we showed that some of this breadth reflects affinity maturation of antibodies within a lineage. This increased activity corresponded to a modest increase in SHM, a central part of the affinity maturation process. For two clonal lineages, the improved breadth was evident not only as expanded neutralization against SARS-CoV-2 variants, including Omicron variants, but also expanded activity against SARS-CoV-1 and related sarbecoviruses, which were notably not known to be a part of the C68 donor’s exposure history.

Detailed analysis of serum-antibody responses has shown that repeated exposure to the same or closely related immunogens induces a boosting of original B cell clones selected during the first exposure, while maturation of new B cell clones is suppressed (61). This effect, known as immune imprinting, decreases as a function of antigenic distance between heterologous exposures (61). Thus, one explanation for our findings of increased breadth in the PVI-2 mAbs compared to PVI-1 mAbs is that the series of exposures and vaccinations this individual received were antigenically similar enough to engage existing antibody clones but distant enough to induce SHM of original clones toward novel immunogens, selecting for antibodies that target broader, more conserved epitopes.

Several remarkable mAbs with broad activity against either SARS-CoV-2 or against sarbecoviruses from animal reservoirs have been isolated from exposed humans (22, 25, 32), but few have broad activity that encompasses both. C68.490 is thus unique in neutralizing across all of these viruses. Moreover, it shows high potency against sarbecovirus strains from different clades, including SARS-CoV-1, similar to the best of recently described antibodies elicited in rabbits in response to a mosaic vaccine expressing diverse sarbecovirus RBD proteins (62). Remarkably, it bound to all 72 sarbecovirus spike RBD proteins tested, including more diverse non-ACE2 utilizing bat sarbecoviruses. Overall, the breadth of C68.490 exceeds that of previously described mAbs with notable activity across sarbecoviruses, including VIR-7229, SA55, and S2X259 (22, 26, 38), as well as C68.61, isolated from this individual after the first breakthrough infection (36). These findings, along with results indicating that escape variants do not evolve under selection by C68.490 in culture, indicate that this mAb targets a highly conserved epitope present in diverse strains, suggesting functional constraints on this epitope.

The C68.490 epitope mapped to the RBD, including positions 378 and 384, which is a more conserved epitope that lends toward greater antibody breadth compared to mAbs that interact with the ACE2-binding surface of the RBD (27, 63). Only a few mutations led to reduced binding of C68.490 to the SARS-CoV-2 spike, implying limited paths to escape. Mutations at positions 378 and 384 were found to reduce binding for both WH-1 and Omicron BA-2 strains, with additional selection at position 386 in the context of the ancestral WH-1 strain. Selected mutations to aspartic acid and glutamic acid were most common, suggesting that a change to an acidic amino acid may be critical for escape. Similarly, the mutations that enabled escape in the context of SARS-CoV-1 spike were primarily aspartic or glutamic acid. However, the escape profile for SARS-CoV-1 included four sites of escape: two that corresponded to those observed for SARS-CoV-2 and two others at nearby sites, 383 and 387. Notably, all of these sites are highly conserved across SARS-CoV-2 and sarbecovirus strains, with occasional mutations at position 384 that were not predicted to impact binding, suggesting C68.490 may bind an evolutionarily constrained epitope.

Monoclonal antibody therapy proved to be a valuable tool for reducing the severity of COVID-19 due to SARS-CoV-2 infection, especially before vaccines were available. However, at the time of this article’s writing, only one antibody therapeutic, PEMGARDA (pemivibart), is authorized by the FDA for emergency use with the condition that the combined national frequency of SARS-CoV-2 variants with substantially reduced susceptibility to PEMGARDA remains less than or equal to 90% (64). Given that there have been multiple sarbecovirus spillover events in recent times, including SARS-CoV-2, which continues to cause significant morbidity and mortality, it is critical to continue to prepare for the future introduction of new sarbecovirus infections in humans, leveraging what we have learned from SARS-CoV-2. Our studies suggest that repeated SARS-CoV-2 exposure with different strains may not only improve responses to that virus, but also elicit broad and potent responses to related viruses present in animal reservoirs. These responses, and the antibodies that drive them, may provide valuable first-line approaches to the next pandemic.

## SUPPLEMENTAL MATERIAL

Supplemental Figures mbio.03612-25-s0001.pdf Fig. S1 and S2.

ASM does not own the copyrights to Supplemental Material that may be linked to, or accessed through, an article. The authors have granted ASM a non-exclusive, world-wide license to publish the Supplemental Material files. Please contact the corresponding author directly for reuse.

## References

[1] Goldblatt D, Alter G, Crotty S, Plotkin SA. 2022. Correlates of protection against SARS-CoV-2 infection and COVID-19 disease. Immunol Rev 310:6–26. doi:10.1111/imr.13091 35661178 PMC9348242

[2] Chen P, Nirula A, Heller B, Gottlieb RL, Boscia J, Morris J, Huhn G, Cardona J, Mocherla B, Stosor V, Shawa I, Adams AC, Van Naarden J, Custer KL, Shen L, Durante M, Oakley G, Schade AE, Sabo J, Patel DR, Klekotka P, Skovronsky DM, BLAZE-1 Investigators. 2021. SARS-CoV-2 neutralizing antibody LY-CoV555 in outpatients with COVID-19. N Engl J Med 384:229–237. doi:10.1056/NEJMoa2029849 33113295 PMC7646625

[3] Copin R, Baum A, Wloga E, Pascal KE, Giordano S, Fulton BO, Zhou A, Negron N, Lanza K, Chan N, et al.. 2021. The monoclonal antibody combination REGEN-COV protects against SARS-COV-2 mutational escape in preclinical and human studies. Cell 184:3949–3961. doi:10.1016/j.cell.2021.06.002 34161776 PMC8179113

[4] Gupta A, Gonzalez-Rojas Y, Juarez E, Crespo Casal M, Moya J, Falci DR, Sarkis E, Solis J, Zheng H, Scott N, Cathcart AL, Hebner CM, Sager J, Mogalian E, Tipple C, Peppercorn A, Alexander E, Pang PS, Free A, Brinson C, Aldinger M, Shapiro AE, COMET-ICE Investigators. 2021. Early treatment for COVID-19 with SARS-CoV-2 neutralizing antibody sotrovimab. N Engl J Med 385:1941–1950. doi:10.1056/NEJMoa2107934 34706189

[5] Weinreich DM, Sivapalasingam S, Norton T, Ali S, Gao H, Bhore R, Xiao J, Hooper AT, Hamilton JD, Musser BJ, et al.. 2021. REGEN-COV antibody combination and outcomes in outpatients with COVID-19. N Engl J Med 385:e81. doi:10.1056/NEJMoa2108163 34587383 PMC8522800

[6] Weinreich DM, Sivapalasingam S, Norton T, Ali S, Gao H, Bhore R, Musser BJ, Soo Y, Rofail D, Im J, et al.. 2021. REGN-COV2, a neutralizing antibody cocktail, in outpatients with COVID-19. N Engl J Med 384:238–251. doi:10.1056/NEJMoa2035002 33332778 PMC7781102

[7] ter Meulen J, van den Brink EN, Poon LLM, Marissen WE, Leung CSW, Cox F, Cheung CY, Bakker AQ, Bogaards JA, van Deventer E, Preiser W, Doerr HW, Chow VT, de Kruif J, Peiris JSM, Goudsmit J. 2006. Human monoclonal antibody combination against SARS coronavirus: synergy and coverage of escape mutants. PLoS Med 3:e237. doi:10.1371/journal.pmed.0030237 16796401 PMC1483912

[8] Wong VWS, Dai D, Wu AKL, Sung JJY. 2003. Treatment of severe acute respiratory syndrome with convalescent plasma. Hong Kong Med J 9:199–201. 12777656

[9] Ge X-Y, Li J-L, Yang X-L, Chmura AA, Zhu G, Epstein JH, Mazet JK, Hu B, Zhang W, Peng C, Zhang Y-J, Luo C-M, Tan B, Wang N, Zhu Y, Crameri G, Zhang S-Y, Wang L-F, Daszak P, Shi Z-L. 2013. Isolation and characterization of a bat SARS-like coronavirus that uses the ACE2 receptor. Nature 503:535–538. doi:10.1038/nature12711 24172901 PMC5389864

[10] Roelle SM, Shukla N, Pham AT, Bruchez AM, Matreyek KA. 2022. Expanded ACE2 dependencies of diverse SARS-like coronavirus receptor binding domains. PLoS Biol 20:e3001738. doi:10.1371/journal.pbio.3001738 35895696 PMC9359572

[11] Seifert SN, Bai S, Fawcett S, Norton EB, Zwezdaryk KJ, Robinson J, Gunn B, Letko M. 2022. An ACE2-dependent sarbecovirus in Russian bats is resistant to SARS-CoV-2 vaccines. PLoS Pathog 18:e1010828. doi:10.1371/journal.ppat.1010828 36136995 PMC9498966

[12] Wacharapluesadee S, Tan CW, Maneeorn P, Duengkae P, Zhu F, Joyjinda Y, Kaewpom T, Chia WN, Ampoot W, Lim BL, Worachotsueptrakun K, Chen V-W, Sirichan N, Ruchisrisarod C, Rodpan A, Noradechanon K, Phaichana T, Jantarat N, Thongnumchaima B, Tu C, Crameri G, Stokes MM, Hemachudha T, Wang L-F. 2021. Evidence for SARS-CoV-2 related coronaviruses circulating in bats and pangolins in Southeast Asia. Nat Commun 12:972. doi:10.1038/s41467-021-21240-1 33563978 PMC7873279

[13] Dejnirattisai W, Zhou D, Ginn HM, Duyvesteyn HME, Supasa P, Case JB, Zhao Y, Walter TS, Mentzer AJ, Liu C, et al.. 2021. The antigenic anatomy of SARS-CoV-2 receptor binding domain. Cell 184:2183–2200. doi:10.1016/j.cell.2021.02.032 33756110 PMC7891125

[14] Greaney AJ, Starr TN, Gilchuk P, Zost SJ, Binshtein E, Loes AN, Hilton SK, Huddleston J, Eguia R, Crawford KHD, Dingens AS, Nargi RS, Sutton RE, Suryadevara N, Rothlauf PW, Liu Z, Whelan SPJ, Carnahan RH, Crowe JE Jr, Bloom JD. 2021. Complete mapping of mutations to the SARS-CoV-2 spike receptor-binding domain that escape antibody recognition. Cell Host Microbe 29:44–57. doi:10.1016/j.chom.2020.11.007 33259788 PMC7676316

[15] Piccoli L, Park Y-J, Tortorici MA, Czudnochowski N, Walls AC, Beltramello M, Silacci-Fregni C, Pinto D, Rosen LE, Bowen JE, et al.. 2020. Mapping neutralizing and immunodominant sites on the SARS-CoV-2 spike receptor-binding domain by structure-guided high-resolution serology. Cell 183:1024–1042. doi:10.1016/j.cell.2020.09.037 32991844 PMC7494283

[16] Qi H, Liu B, Wang X, Zhang L. 2022. The humoral response and antibodies against SARS-CoV-2 infection. Nat Immunol 23:1008–1020. doi:10.1038/s41590-022-01248-5 35761083

[17] Robbiani DF, Gaebler C, Muecksch F, Lorenzi JCC, Wang Z, Cho A, Agudelo M, Barnes CO, Gazumyan A, Finkin S, et al.. 2020. Convergent antibody responses to SARS-CoV-2 in convalescent individuals. Nature 584:437–442. doi:10.1038/s41586-020-2456-9 32555388 PMC7442695

[18] Cao Y, Jian F, Wang J, Yu Y, Song W, Yisimayi A, Wang J, An R, Chen X, Zhang N, et al.. 2023. Imprinted SARS-CoV-2 humoral immunity induces convergent Omicron RBD evolution. Nature 614:521–529. doi:10.1038/s41586-022-05644-7 36535326 PMC9931576

[19] Starr TN, Greaney AJ, Hilton SK, Ellis D, Crawford KHD, Dingens AS, Navarro MJ, Bowen JE, Tortorici MA, Walls AC, King NP, Veesler D, Bloom JD. 2020. Deep mutational scanning of SARS-CoV-2 receptor binding domain reveals constraints on folding and ACE2 binding. Cell 182:1295–1310. doi:10.1016/j.cell.2020.08.012 32841599 PMC7418704

[20] Taylor AL, Starr TN. 2024. Deep mutational scanning of SARS-CoV-2 Omicron BA.2.86 and epistatic emergence of the KP.3 variant. Virus Evol 10:veae067. doi:10.1093/ve/veae067 39310091 PMC11414647

[21] Witte L, Baharani VA, Schmidt F, Wang Z, Cho A, Raspe R, Guzman-Cardozo C, Muecksch F, Canis M, Park DJ, Gaebler C, Caskey M, Nussenzweig MC, Hatziioannou T, Bieniasz PD. 2023. Epistasis lowers the genetic barrier to SARS-CoV-2 neutralizing antibody escape. Nat Commun 14:302. doi:10.1038/s41467-023-35927-0 36653360 PMC9849103

[22] Cameroni E, Bowen JE, Rosen LE, Saliba C, Zepeda SK, Culap K, Pinto D, VanBlargan LA, De Marco A, di Iulio J, et al.. 2022. Broadly neutralizing antibodies overcome SARS-CoV-2 Omicron antigenic shift. Nature 602:664–670. doi:10.1038/s41586-021-04386-2 35016195 PMC9531318

[23] Cao Y, Wang J, Jian F, Xiao T, Song W, Yisimayi A, Huang W, Li Q, Wang P, An R, et al.. 2022. Omicron escapes the majority of existing SARS-CoV-2 neutralizing antibodies. Nature 602:657–663. doi:10.1038/s41586-021-04385-3 35016194 PMC8866119

[24] Cao Y, Yisimayi A, Jian F, Song W, Xiao T, Wang L, Du S, Wang J, Li Q, Chen X, et al.. 2022. BA.2.12.1, BA.4 and BA.5 escape antibodies elicited by Omicron infection. Nature 608:593–602. doi:10.1038/s41586-022-04980-y 35714668 PMC9385493

[25] Tortorici MA, Czudnochowski N, Starr TN, Marzi R, Walls AC, Zatta F, Bowen JE, Jaconi S, Di Iulio J, Wang Z, et al.. 2021. Broad sarbecovirus neutralization by a human monoclonal antibody. Nature 597:103–108. doi:10.1038/s41586-021-03817-4 34280951 PMC9341430

[26] Cao Y, Jian F, Zhang Z, Yisimayi A, Hao X, Bao L, Yuan F, Yu Y, Du S, Wang J, et al.. 2022. Rational identification of potent and broad sarbecovirus-neutralizing antibody cocktails from SARS convalescents. Cell Rep 41:111845. doi:10.1016/j.celrep.2022.111845 36493787 PMC9712074

[27] Starr TN, Czudnochowski N, Liu Z, Zatta F, Park Y-J, Addetia A, Pinto D, Beltramello M, Hernandez P, Greaney AJ, et al.. 2021. SARS-CoV-2 RBD antibodies that maximize breadth and resistance to escape. Nature 597:97–102. doi:10.1038/s41586-021-03807-6 34261126 PMC9282883

[28] Cui L, Li T, Lan M, Zhou M, Xue W, Zhang S, Wang H, Hong M, Zhang Y, Yuan L, Sun H, Ye J, Zheng Q, Guan Y, Gu Y, Xia N, Li S. 2024. A cryptic site in class 5 epitope of SARS-CoV-2 RBD maintains highly conservation across natural isolates. iScience 27:110208. doi:10.1016/j.isci.2024.110208 39015149 PMC11251093

[29] Park Y-J, De Marco A, Starr TN, Liu Z, Pinto D, Walls AC, Zatta F, Zepeda SK, Bowen JE, Sprouse KR, et al.. 2022. Antibody-mediated broad sarbecovirus neutralization through ACE2 molecular mimicry. Science 375:449–454. doi:10.1126/science.abm8143 34990214 PMC9400459

[30] Rappazzo CG, Tse LV, Kaku CI, Wrapp D, Sakharkar M, Huang D, Deveau LM, Yockachonis TJ, Herbert AS, Battles MB, et al.. 2021. Broad and potent activity against SARS-like viruses by an engineered human monoclonal antibody. Science 371:823–829. doi:10.1126/science.abf4830 33495307 PMC7963221

[31] Jensen JL, Sankhala RS, Dussupt V, Bai H, Hajduczki A, Lal KG, Chang WC, Martinez EJ, Peterson CE, Golub ES, et al.. 2023. Targeting the spike receptor binding domain class V cryptic epitope by an antibody with pan-sarbecovirus activity. J Virol 97:e01596-22. doi:10.1128/jvi.01596-22 37395646 PMC10373542

[32] Pinto D, Park Y-J, Beltramello M, Walls AC, Tortorici MA, Bianchi S, Jaconi S, Culap K, Zatta F, De Marco A, Peter A, Guarino B, Spreafico R, Cameroni E, Case JB, Chen RE, Havenar-Daughton C, Snell G, Telenti A, Virgin HW, Lanzavecchia A, Diamond MS, Fink K, Veesler D, Corti D. 2020. Cross-neutralization of SARS-CoV-2 by a human monoclonal SARS-CoV antibody. Nature 583:290–295. doi:10.1038/s41586-020-2349-y 32422645

[33] Jette CA, Cohen AA, Gnanapragasam PNP, Muecksch F, Lee YE, Huey-Tubman KE, Schmidt F, Hatziioannou T, Bieniasz PD, Nussenzweig MC, West AP, Keeffe JR, Bjorkman PJ, Barnes CO. 2021. Broad cross-reactivity across sarbecoviruses exhibited by a subset of COVID-19 donor-derived neutralizing antibodies. Cell Rep 36:109760. doi:10.1016/j.celrep.2021.109760 34534459 PMC8423902

[34] Liu H, Wu NC, Yuan M, Bangaru S, Torres JL, Caniels TG, van Schooten J, Zhu X, Lee C-CD, Brouwer PJM, van Gils MJ, Sanders RW, Ward AB, Wilson IA. 2020. Cross-neutralization of a SARS-CoV-2 antibody to a functionally conserved site is mediated by avidity. Immunity 53:1272–1280. doi:10.1016/j.immuni.2020.10.023 33242394 PMC7687367

[35] Huang K-YA, Chen X, Mohapatra A, Nguyen HTV, Schimanski L, Tan TK, Rijal P, Vester SK, Hills RA, Howarth M, Keeffe JR, Cohen AA, Kakutani LM, Wu Y-M, Shahed-Al-Mahmud M, Chou Y-C, Bjorkman PJ, Townsend AR, Ma C. 2023. Structural basis for a conserved neutralization epitope on the receptor-binding domain of SARS-CoV-2. Nat Commun 14:311. doi:10.1038/s41467-023-35949-8 36658148 PMC9852238

[36] Guenthoer J, Lilly M, Starr TN, Dadonaite B, Lovendahl KN, Croft JT, Stoddard CI, Chohan V, Ding S, Ruiz F, Kopp MS, Finzi A, Bloom JD, Chu HY, Lee KK, Overbaugh J. 2023. Identification of broad, potent antibodies to functionally constrained regions of SARS-CoV-2 spike following a breakthrough infection. Proc Natl Acad Sci USA 120. doi:10.1073/pnas.2220948120 PMC10265947 37253011

[37] Ruiz F, Foreman WB, Lilly M, Baharani VA, Depierreux DM, Chohan V, Taylor AL, Guenthoer J, Ralph D, Matsen IV FA, Chu HY, Bieniasz PD, Côté M, Starr TN, Overbaugh J. 2024. Delineating the functional activity of antibodies with cross-reactivity to SARS-CoV-2, SARS-CoV-1 and related sarbecoviruses. PLoS Pathog 20:e1012650. doi:10.1371/journal.ppat.1012650 39466880 PMC11542851

[38] Rosen LE, Tortorici MA, De Marco A, Pinto D, Foreman WB, Taylor AL, Park Y-J, Bohan D, Rietz T, Errico JM, et al.. 2024. A potent pan-sarbecovirus neutralizing antibody resilient to epitope diversification. Cell 187:7196–7213. doi:10.1016/j.cell.2024.09.026 39383863 PMC11645210

[39] Planas D, Staropoli I, Planchais C, Yab E, Jeyarajah B, Rahou Y, Prot M, Guivel-Benhassine F, Lemoine F, Enouf V, Simon-Loriere E, Mouquet H, Rameix-Welti M-A, Schwartz O. 2024. Escape of SARS-CoV-2 Variants KP.1.1, LB.1, and KP3.3 from approved monoclonal antibodies. Pathog Immun 10:1–11. doi:10.20411/pai.v10i1.752 39391808 PMC11464000

[40] Wang Q, Bowen A, Ho J, Zhang RM, Valdez R, Stoneman E, Gordon A, Liu L, Ho DD. 2023. SARS-CoV-2 neutralising antibodies after a second BA.5 bivalent booster. Lancet 402:1827–1828. doi:10.1016/S0140-6736(23)02278-X 37922920 PMC13141638

[41] Wang Q, Guo Y, Bowen A, Mellis IA, Valdez R, Gherasim C, Gordon A, Liu L, Ho DD. 2024. XBB.1.5 monovalent mRNA vaccine booster elicits robust neutralizing antibodies against XBB subvariants and JN.1. Cell Host Microbe 32:315–321. doi:10.1016/j.chom.2024.01.014 38377995 PMC10948033

[42] Abbad A, Yellin T, Singh G, Fried M, Raskin A, Tcheou J, Monahan B, Gleason C, Andre D, Bermúdez-González MC, et al.. 2024. SARS-CoV-2 BA.1 and BA.2 breakthrough infections boost antibody responses to early Omicron subvariants but not BQ.1.1 or XBB.1.5. Cell Rep Med 5:101474. doi:10.1016/j.xcrm.2024.101474 38508136 PMC10983110

[43] Evans JP, Zeng C, Carlin C, Lozanski G, Saif LJ, Oltz EM, Gumina RJ, Liu S-L. 2022. Neutralizing antibody responses elicited by SARS-CoV-2 mRNA vaccination wane over time and are boosted by breakthrough infection. Sci Transl Med 14:eabn8057. doi:10.1126/scitranslmed.abn8057 35166573 PMC8939766

[44] Kaku CI, Starr TN, Zhou P, Dugan HL, Khalifé P, Song G, Champney ER, Mielcarz DW, Geoghegan JC, Burton DR, Andrabi R, Bloom JD, Walker LM. 2023. Evolution of antibody immunity following Omicron BA.1 breakthrough infection. Nat Commun 14:2751. doi:10.1038/s41467-023-38345-4 37173311 PMC10180619

[45] Kotaki R, Moriyama S, Oishi S, Onodera T, Adachi Y, Sasaki E, Ishino K, Morikawa M, Takei H, Takahashi H, Takano T, Nishiyama A, Yumoto K, Terahara K, Isogawa M, Matsumura T, Shinkai M, Takahashi Y. 2024. Repeated Omicron exposures redirect SARS-CoV-2-specific memory B cell evolution toward the latest variants. Sci Transl Med 16:eadp9927. doi:10.1126/scitranslmed.adp9927 39167666

[46] Kurhade C, Zou J, Xia H, Liu M, Chang HC, Ren P, Xie X, Shi P-Y. 2023. Low neutralization of SARS-CoV-2 Omicron BA.2.75.2, BQ.1.1 and XBB.1 by parental mRNA vaccine or a BA.5 bivalent booster. Nat Med 29:344–347. doi:10.1038/s41591-022-02162-x 36473500

[47] Collier A-RY, Miller J, Hachmann NP, McMahan K, Liu J, Bondzie EA, Gallup L, Rowe M, Schonberg E, Thai S, Barrett J, Borducchi EN, Bouffard E, Jacob-Dolan C, Mazurek CR, Mutoni A, Powers O, Sciacca M, Surve N, VanWyk H, Wu C, Barouch DH. 2023. Immunogenicity of BA.5 bivalent MRNA Vaccine boosters. N Engl J Med 388:565–567. doi:10.1056/NEJMc2213948 36630611 PMC9847505

[48] Quandt J, Muik A, Salisch N, Lui BG, Lutz S, Krüger K, Wallisch A-K, Adams-Quack P, Bacher M, Finlayson A, Ozhelvaci O, Vogler I, Grikscheit K, Hoehl S, Goetsch U, Ciesek S, Türeci Ö, Sahin U. 2022. Omicron BA.1 breakthrough infection drives cross-variant neutralization and memory B cell formation against conserved epitopes. Sci Immunol 7:eabq2427. doi:10.1126/sciimmunol.abq2427 35653438 PMC9162083

[49] Guenthoer J, Garrett ME, Lilly M, Depierreux DM, Ruiz F, Chi M, Stoddard CI, Chohan V, Yaffe ZA, Sung K, Ralph D, Chu HY, Matsen FA, Overbaugh J. 2024. The S2 subunit of spike encodes diverse targets for functional antibody responses to SARS-CoV-2. PLoS Pathog 20:e1012383. doi:10.1371/journal.ppat.1012383 39093891 PMC11324185

[50] Tiller T, Meffre E, Yurasov S, Tsuiji M, Nussenzweig MC, Wardemann H. 2008. Efficient generation of monoclonal antibodies from single human B cells by single cell RT-PCR and expression vector cloning. J Immunol Methods 329:112–124. doi:10.1016/j.jim.2007.09.017 17996249 PMC2243222

[51] Huang J, Doria-Rose NA, Longo NS, Laub L, Lin C-L, Turk E, Kang BH, Migueles SA, Bailer RT, Mascola JR, Connors M. 2013. Isolation of human monoclonal antibodies from peripheral blood B cells. Nat Protoc 8:1907–1915. doi:10.1038/nprot.2013.117 24030440 PMC4844175

[52] Ralph DK, Matsen FA. 2016. Consistency of VDJ rearrangement and substitution parameters enables accurate B cell receptor sequence annotation. PLoS Comput Biol 12:e1004409. doi:10.1371/journal.pcbi.1004409 26751373 PMC4709141

[53] Ralph DK, Matsen FA. 2022. Inference of B cell clonal families using heavy/light chain pairing information. PLoS Comput Biol 18:e1010723. doi:10.1371/journal.pcbi.1010723 36441808 PMC9731466

[54] Garrett ME, Galloway J, Chu HY, Itell HL, Stoddard CI, Wolf CR, Logue JK, McDonald D, Weight H, Matsen FA IV, Overbaugh J. 2021. High-resolution profiling of pathways of escape for SARS-CoV-2 spike-binding antibodies. Cell 184:2927–2938. doi:10.1016/j.cell.2021.04.045 34010620 PMC8096189

[55] Crawford KHD, Eguia R, Dingens AS, Loes AN, Malone KD, Wolf CR, Chu HY, Tortorici MA, Veesler D, Murphy M, Pettie D, King NP, Balazs AB, Bloom JD. 2020. Protocol and reagents for pseudotyping lentiviral particles with SARS-CoV-2 spike protein for neutralization assays. Viruses 12:513. doi:10.3390/v12050513 32384820 PMC7291041

[56] Starr TN, Zepeda SK, Walls AC, Greaney AJ, Alkhovsky S, Veesler D, Bloom JD. 2022. ACE2 binding is an ancestral and evolvable trait of sarbecoviruses. Nature 603:913–918. doi:10.1038/s41586-022-04464-z 35114688 PMC8967715

[57] Dingens AS, Arenz D, Weight H, Overbaugh J, Bloom JD. 2019. An antigenic atlas of HIV-1 escape from broadly neutralizing antibodies distinguishes functional and structural epitopes. Immunity 50:520–532. doi:10.1016/j.immuni.2018.12.017 30709739 PMC6435357

[58] Starr TN, Greaney AJ, Stewart CM, Walls AC, Hannon WW, Veesler D, Bloom JD. 2022. Deep mutational scans for ACE2 binding, RBD expression, and antibody escape in the SARS-CoV-2 Omicron BA.1 and BA.2 receptor-binding domains. PLoS Pathog 18:e1010951. doi:10.1371/journal.ppat.1010951 36399443 PMC9674177

[59] Lee J, Zepeda SK, Park Y-J, Taylor AL, Quispe J, Stewart C, Leaf EM, Treichel C, Corti D, King NP, Starr TN, Veesler D. 2023. Broad receptor tropism and immunogenicity of a clade 3 sarbecovirus. Cell Host Microbe 31:1961–1973. doi:10.1016/j.chom.2023.10.018 37989312 PMC10913562

[60] Hadfield J, Megill C, Bell SM, Huddleston J, Potter B, Callender C, Sagulenko P, Bedford T, Neher RA. 2018. Nextstrain: real-time tracking of pathogen evolution. Bioinformatics 34:4121–4123. doi:10.1093/bioinformatics/bty407 29790939 PMC6247931

[61] Schiepers A, van ’t Wout MFL, Greaney AJ, Zang T, Muramatsu H, Lin PJC, Tam YK, Mesin L, Starr TN, Bieniasz PD, Pardi N, Bloom JD, Victora GD. 2023. Molecular fate-mapping of serum antibody responses to repeat immunization. Nature 615:482–489. doi:10.1038/s41586-023-05715-3 36646114 PMC10023323

[62] Fan C, Keeffe JR, Malecek KE, Cohen AA, West AP, Baharani VA, Rorick AV, Gao H, Gnanapragasam PNP, Rho S, Alvarez J, Segovia LN, Hatziioannou T, Bieniasz PD, Bjorkman PJ. 2025. Cross-reactive sarbecovirus antibodies induced by mosaic RBD nanoparticles. Proc Natl Acad Sci USA 122:e2501637122. doi:10.1073/pnas.2501637122 40402246 PMC12130868

[63] Fan C, Cohen AA, Park M, Hung AF-H, Keeffe JR, Gnanapragasam PNP, Lee YE, Gao H, Kakutani LM, Wu Z, Kleanthous H, Malecek KE, Williams JC, Bjorkman PJ. 2022. Neutralizing monoclonal antibodies elicited by mosaic RBD nanoparticles bind conserved sarbecovirus epitopes. Immunity 55:2419–2435. doi:10.1016/j.immuni.2022.10.019 36370711 PMC9606073

[64] Cavazzoni PA. 2024. Emergency use authorization 122. Available from: https://www.fda.gov/media/177068/download

