# Supplementary material for: Re-infection with SARS-CoV-2 is associated with increased antibody breadth and potency against diverse sarbecovirus strains
Source: mBio. 2026 Feb 25;17(4):e03612-25. doi: 10.1128/mbio.03612-25 (PMC13059721; doi:10.1128/mbio.03612-25)
Supplement: Supplemental Figures — Fig. S1 and S2. [file mbio.03612-25-s0001.pdf]

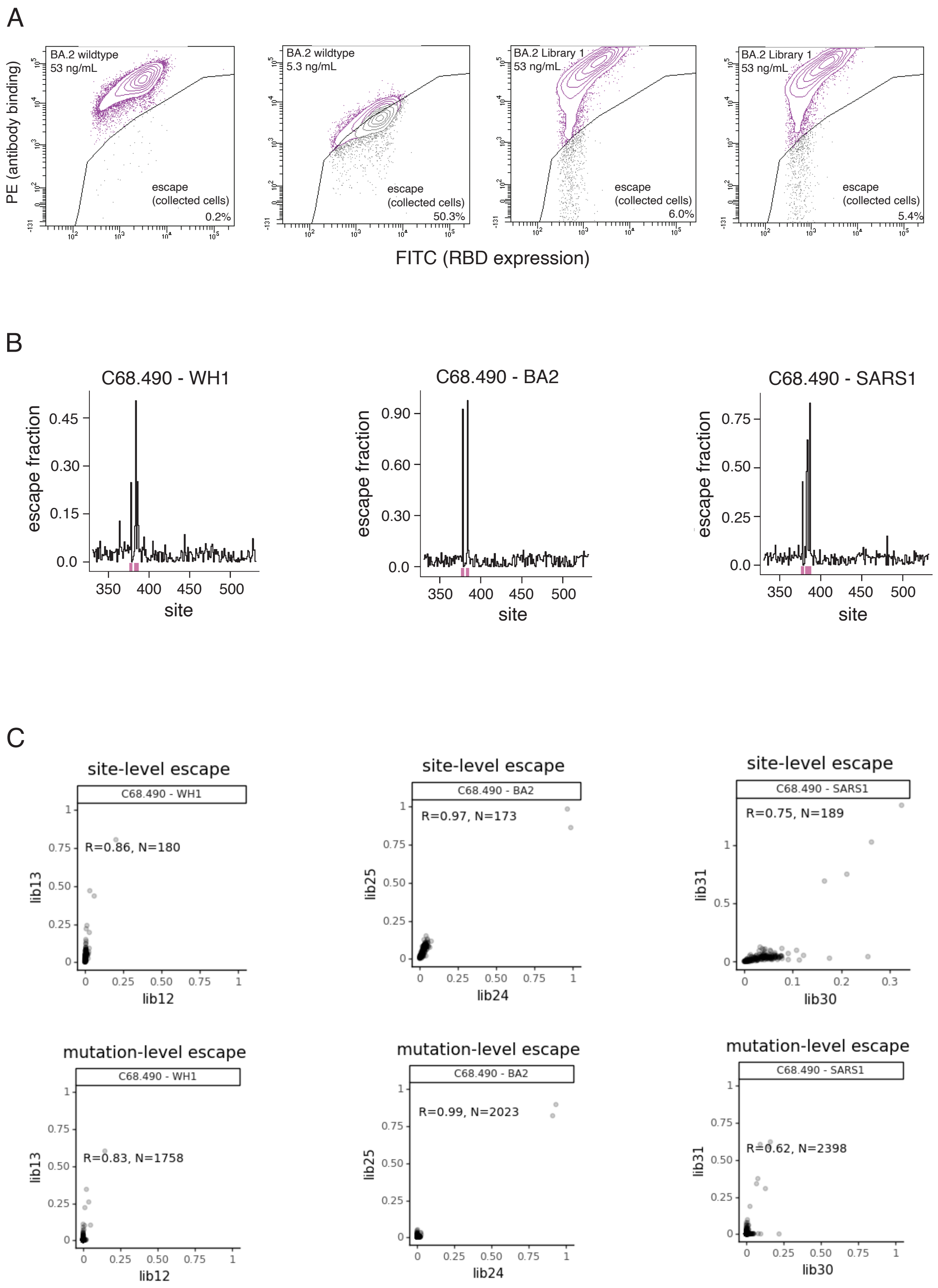

Supplemental Figure 1. FACS Gating scheme, line plots of escape per site, and correlations of escape across replicates for yeast-display DMS escape selections.

			Heavy Chain					Light Chain			
Clonal Family	mAb	Timepoint	V-gene	D-gene	J-gene	CDR3 Length	% SHM	V-gene	J-gene	CDR3 Length	% SHM
1	C68.285	PVI-1	IGHV1-46	IGHD5-24	IGHJ4	13	2.3%	IGKV1-9	IGKJ4	13	0.9%
	C68.696	**PVI-2**					3.4%				1.8%
2	C68.10	PVI-1	IGHV5-51	IGHD3-3	IGHJ4	14	3.4%	IGLV3-19	IGLJ2	13	1.6%
	C68.560	**PVI-2**					3.6%				2.3%
3	C68.83	PVI-1	IGHV3-30	IGHD2-15	IGHJ6	24	2.8%	IGLV3-25	IGLJ2	13	4.6%
	C68.699	**PVI-2**					3.9%				2.9%
4	C68.200	PVI-1	IGH3-23	IGHD1-1	IGHJ4	15	3.1%	IGKV1-5	IGKJ5	10	0.6%
	C68.203	PVI-1					3.1%				1.3%
	C68.720	**PVI-2**					10.3%				1.6%
5	C68.459	**PVI-2**	IGHV4-31	IGHD5-5	IGH4	13	8.7%	IGKV4-1	IGKJ2	11	3.2%
6	C68.470	**PVI-2**	IGHV5-10-1	IGHD4-17	IGH4	17	5.5%	IGLV2-23	IGLJ3	10	4.0%
	C68.773	**PVI-2**					4.6%				2.9%
7	C68.490	**PVI-2**	IGHV1-18	IGHD1-26	IGHJ3	24	6.2%	IGKV1-5	IGKJ4	11	3.4%
8	C68.586	**PVI-2**	IGHV5-51	IGHD2-21	IGHJ4	13	7.2%	IGKV1-5	IGKJ1	11	2.8%
9	C68.571	**PVI-2**	IGHV4-59	IGHD4-23	IGHJ4	15	8.1%	IGKV1-33	IGKJ4	11	5.0%
10	C68.554	**PVI-2**	IGHV5-51	IGHD2-21	IGH4	15	7.8%	IGKV1-5	IGKJ1	11	4.4%
11	C68.654	**PVI-2**	IGHV3-64D	IGHD5-24	IGHJ3	18	4.9%	IGKV1D-39	IGKJ4	10	1.6%
12	C68.715	**PVI-2**	IGH3-23	IGHD1-1	IGH4	15	6.1%	IGKV1-5	IGKJ1	11	0.6%
13	C68.685	**PVI-2**	IGH3-23	IGHD2-15	IGH4	15	7.5%	IGHK1-5	IGKJ2	11	1.9%
	C68.757	**PVI-2**					8.9%				3.1%

Supplemental Figure 2. Gene Family usage for all RBD clonal antibodies
